# Treatment and Pathology of Gout

**Published:** 1881-06

**Authors:** 


TREATMENT AND PATHOLOGY OF GOUT.

MELDON in a recent communication (*British Medical Journal,* March 26, 1881,) comes to the following conclusions respecting gout: First, that the presence of uric acid and soda in the blood is not the sole cause of gout. Second, want of exercise, and animal diet, will cause an accumulation of uric acid in the blood. Third, uric acid and soda must exist in the blood before the disease can be produced. Fourth, there must be depression of the nervous system to cause an attack of gout. Fifth, depression of the nervous system causes a union of uric acid and soda, forming urate of soda. Sixth, when an attack of gout has passed away, it does not necessarily follow that uric acid has disappeared from the blood. Seventh, uric acid may exist in the blood in considerable quantities and for any length of time, without causing gout. Eighth, the use of nerve tonics like quinine, strychnia, caffeine, and such like, as well as the inhaling of oxygen, are of much service in the treatment of this disease. The most striking of these conclusions is the fifth, which borders on the almost purely hypothetical, as no experimental or clinical evidence of this view has as yet been reported.

